# Note from the Editor: New Author Instructions

**Published:** 2008-10

*EHP* has revised its Instructions to Authors. The new version became available on the journal’s website on 19 September 2008, and a print version is provided on page A444. The instructions now provide background information (Who We Are) and describe the type of multidisciplinary research of interest to the journal (What We Publish). Sections are organized to address the various phases of publication (e.g., About Your Manuscript, Manuscript Preparation, Manuscript Submission, Publication Sequence). The instructions also include a section on the publication style preferred by the journal (*EHP* Style).

Please note that the journal remains committed to the full disclosure of all potential competing interests for authors, reviewers, and *EHP* editors. For the first time, we have described *EHP* ’s policy concerning potential nonfinancial conflicts of interest. This policy pertains primarily to reviewers and editors.

The new Instructions to Authors also describes in considerable detail the process for using the new Manuscript Central (ScholarOne) tracking system that we announced in the August issue of the journal [Environ Health Perspect 116:A328 (2008)]. New manuscripts should be submitted using this system (http://mc.manuscriptcentral.com/ehp). Authors should also note the new page charges and costs for color figures effective 1 August 2008.

